# A synthetic probiotic engineered for colorectal cancer therapy modulates gut microbiota

**DOI:** 10.1186/s40168-021-01071-4

**Published:** 2021-05-26

**Authors:** Yusook Chung, Yongku Ryu, Byung Chull An, Yeo-Sang Yoon, Oksik Choi, Tai Yeub Kim, Jaekyung Yoon, Jun Young Ahn, Ho Jin Park, Soon-Kyeong Kwon, Jihyun F. Kim, Myung Jun Chung

**Affiliations:** 1 R&D Center, Cell Biotech, Co., Ltd., 50 Aegibong-ro 409beon-gil, Gaegok-ri, Wolgot-myeon, Gimpo-si, Gyeonggi-do 10003 Republic of Korea; 2 Department of Systems Biology, Division of Life Sciences, and Institute for Life Science and Biotechnology, Yonsei University, 50 Yonsei-ro, Seodaemun-gu, Seoul, 03722 Republic of Korea; 3 Division of Applied Life Science (BK21), Gyeongsang National University, 501 Jinju-daero, Jinju-si, Gyeongsangnam-do 52828 Republic of Korea

**Keywords:** *Lactobacillus rhamnosus* CBT LR5 (KCTC 12202BP), Alanine racemase, DLD-1 xenograft, AOM/DSS model of colitis-associated cancer, Microbiome, *Akkermansia*, *Turicibacter*

## Abstract

**Background:**

Successful chemoprevention or chemotherapy is achieved through targeted delivery of prophylactic agents during initial phases of carcinogenesis or therapeutic agents to malignant tumors. Bacteria can be used as anticancer agents, but efforts to utilize attenuated pathogenic bacteria suffer from the risk of toxicity or infection. Lactic acid bacteria are safe to eat and often confer health benefits, making them ideal candidates for live vehicles engineered to deliver anticancer drugs.

**Results:**

In this study, we developed an effective bacterial drug delivery system for colorectal cancer (CRC) therapy using the lactic acid bacterium *Pediococcus pentosaceus*. It is equipped with dual gene cassettes driven by a strong inducible promoter that encode the therapeutic protein P8 fused to a secretion signal peptide and a complementation system. In an inducible CRC cell-derived xenograft mouse model, our synthetic probiotic significantly reduced tumor volume and inhibited tumor growth relative to the control. Mice with colitis-associated CRC induced by azoxymethane and dextran sodium sulfate exhibited polyp regression and recovered taxonomic diversity when the engineered bacterium was orally administered. Further, the synthetic probiotic modulated gut microbiota and alleviated the chemically induced dysbiosis. Correlation analysis demonstrated that specific bacterial taxa potentially associated with eubiosis or dysbiosis, such as *Akkermansia* or *Turicibacter*, have positive or negative relationships with other microbial members.

**Conclusions:**

Taken together, our work illustrates that an effective and stable synthetic probiotic composed of *P. pentosaceus* and the P8 therapeutic protein can reduce CRC and contribute to rebiosis, and the validity and feasibility of cell-based designer biopharmaceuticals for both treating CRC and ameliorating impaired microbiota.

Video abstract

**Supplementary Information:**

The online version contains supplementary material available at 10.1186/s40168-021-01071-4.

## Background

Cancer is the leading cause of death in humans and the global burden is rising [1]. Cancer treatments include surgery, chemotherapy, radiotherapy, and targeted therapy. In chemotherapy, natural, synthetic, or biological substances are used as treatments that suppress or prevent cancer progression [2]. However, most chemotherapeutic agents target rapidly dividing cells, which not only include cancer, but also bone marrow or hair follicles as off-target effects. Moreover, drug resistance diminishes the efficacy of chemotherapy and is responsible for the high relapse rate even after successful recovery. Targeted therapy commonly uses biopharmaceuticals that are more specific, less toxic, and rarely cause side effects; however, the selectivity is often insufficient in practice [3, 4].

Bacteria can be utilized to treat cancer and their recognition as anticancer agents dates back more than a century [5]. Strains of potentially harmful or pathogenic bacteria like *Clostridium*, *Listeria*, or *Salmonella* that are either natural, mutated, or genetically modified have been used in cancer therapy due to their ability to colonize the solid tumor under hypoxic conditions and induce tumor shrinkage [6]. Although there have been efforts to make use of attenuated bacteria, the risk for toxicity or infection hampers their clinical applications. Generally recognized as safe and often with health benefits for the host, lactic acid bacteria (LABs) such as *Lactobacillus*, *Lactococcus*, *Leuconostoc*, or *Pediococcus*, are ideal candidates for bacterial therapy [7, 8]. Furthermore, they can be used as live vehicles engineered to deliver anticancer drugs [9].

Colorectal cancer (CRC) is a severe cancer responsible for almost 900,000 deaths annually [10]. A small protein called P8 with a molecular mass of 8 kDa was isolated from *Lactobacillus rhamnosus* CBT LR5, in an effort to screen for novel therapeutic proteins against CRC [11]. To design and develop a clinically relevant system that can be orally administered and still stable, we used an LAB strain, *Pediococcus pentosaceus* SL4 [12], as a safe drug delivery vehicle that expresses and secretes P8 and, thus, avoids its degradation in the gastrointestinal tract. We isolated the *P. pentosaceus* SL4 bacterium from a Korean fermented vegetable food, kimchi, which produces a bacteriocin and inhibits the growth of *Listeria monocytogenes* and *Staphylococcus aureus* [13]*. P. pentosaceus* is Gram-positive, facultatively anaerobic, acid-tolerant, non-motile, and non-spore-forming [12]. It is frequently isolated from fermented foods and applied as the starter culture in dairy or plant fermentation [14, 15]. Strains of this species were able to alleviate azoxymethane-induced toxicity, inhibit colon cancer cell proliferation, and secrete antimicrobial peptides that inhibit pathogenic bacteria [16–18].

In this study, we developed an advanced anti-CRC therapeutic probiotic that utilizes P8 and demonstrated its powerful efficacy using two different murine models: DLD-1 xenograft and colitis-associated tumorigenesis induced by azoxymethane (AOM) and dextran sodium sulfate (DSS). Moreover, considering the significant relationship between gut microbiota and drug response [19], we longitudinally investigated microbiota profiles during the administration of the P8-producing synthetic probiotic in the AOM/DSS model to reveal the complex interactions between individual microbial members and to identify key taxa associated with the use of our synthetic probiotic and AOM/DSS-induced tumorigenesis.

## Results

### Designing an effective drug delivery system for CRC therapy

**Fig. 1:**
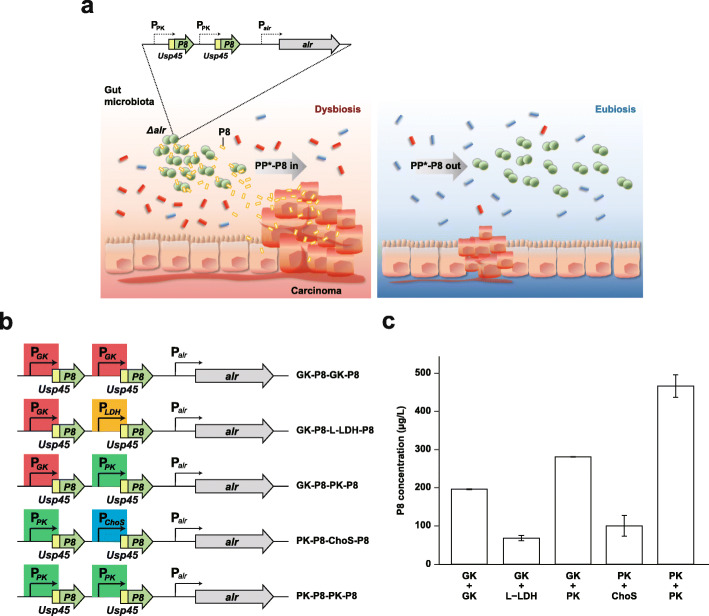
Module design of a lactic acid bacterium-based drug delivery system for optimal P8 productivity. **a** A schematic outline depicting the expected mode of action of the synthetic probiotic PP*-P8 with the *alr* complementation system. *alr*, the alanine racemase gene. **b** Constructs with various promoters for dual expression of the P8 therapeutic protein fused to the 27-residue Usp45 leader peptide. GK, glucose kinase; LDH, l-lactate dehydrogenase; PK, pyruvate kinase; ChoS, choline ABC transporter permease and substrate binding protein. **c** Concentrations of P8 secreted from PP*-P8 that were quantified using ELISA, indicating that the PK-PK promotor system had the highest amount of secreted P8

Figure 1a depicts the design principle of a synthetic probiotic that employs the P8 therapeutic protein to treat or prevent CRC. To design an anti-CRC therapeutic probiotic with enhanced stability and efficacy, we first adopted the *alr* complementation system that can prevent curing of the P8 expression vector, pCBT24-2 [11], in the absence of an antibiotic to maintain the plasmid. Alanine racemase is a pyridoxal 5′-phosphate-dependent enzyme involved in the interconversion of d-alanine (d-Ala) and l-alanine. d-Ala is involved in the cross-linking of the cell wall peptidoglycan layer and exists in extremely low amounts in nature. Thus, this component is essential for bacterial growth and deletion of the *alr* gene leads to cell death. To generate a d-Ala auxotroph of *P. pentosaceus* SL4(-7) that is a derivative of SL4 lacking all seven native plasmids, we performed knockout mutagenesis to remove *alr* from the chromosome using homologous recombination with a construct that has an in-frame deletion of the *alr* gene and 1 kb of its upstream or downstream flanking sequences (Additional file 1, Fig. S1a,b). The resulting auxotrophic mutant was either grown in a medium supplemented with d-Ala or complemented with a plasmid that expresses *alr*. Genotyping with specific primers confirmed the replacement of the intact gene (Additional file 1, Fig. S1c, Additional file 2, Table S1). This *alr* auxotroph complemented with plasmid-borne *alr* was designated as PP*.

In order to develop an effective gene expression system that can maximize the productivity of P8, four kinds of constitutive promoters involved in central glycolytic pathway were selected: pyruvate kinase (PK), choline ABC transporter permease and substrate-binding protein, glucose kinase, and l-lactate dehydrogenase. Using these promoters, we constructed five sets of dual expression systems that have two chimeric genes, each encoding the P8 peptide fused to the Usp45 secretion signal at its N terminus, which was cloned into the vector that contained *alr* (Fig. 1b). We then measured the concentrations of secreted P8 for each PP* clone with the dual expression module in the *alr* vector using ELISA to validate the PK-PK promotor system with the best stability and productivity (Fig. 1c). To further exclude the possibility that the host genotype could affect the performance of P8 secretion, we checked the concentrations of P8 secreted from the wild-type *P. pentosaceus* SL4(-7) with the PK-PK promotor system in pCBT24-2 (PP-P8) and the *Δalr* mutant with the PK-PK promotor system in the *alr* vector (PP*-P8) and found no difference between the SL4 wild type and *Δalr* mutant (Additional file 1, Fig. S1d).

### Anti-tumor efficacy of PP*-P8 in the DLD-1 xenograft mouse model

**Fig. 2:**
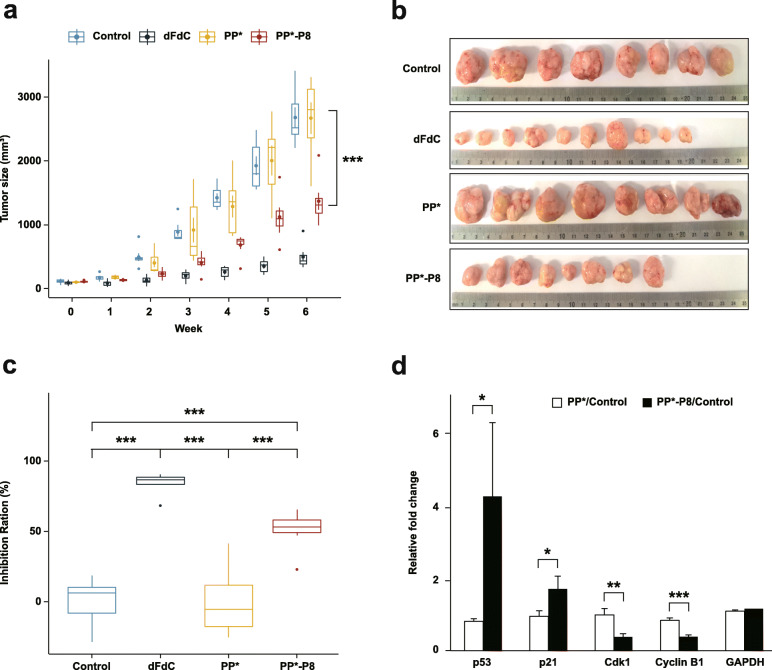
Anti-tumor efficacy of the PP*-P8 probiotic in the DLD-1 xenograft mouse model. **a** Increased sizes of DLD-1-derived tumors recorded each week. Mice (*n* = 10 in each group) were subcutaneously inoculated with 2 × 10^6^ DLD-1 cells in the rear right flank and then received 0.9% saline (control), 60 mg/kg body weight gemcitabine (dFdC; intraperitoneal injection, twice a week), 1 × 10^10^ CFU/head *P. pentosaceus alr* (pCBT24-2-alr) (PP*; oral administration, five times a week), or 1 × 10^10^ CFU/head *P. pentosaceus alr* (pCBT24-2-PK-p8-PK-p8-alr) (PP*-P8; oral administration, five times a week). ***P < 0.001 for control vs. dFdC, control vs. PP*-P8, dFdC vs. PP*, PP* vs. PP*-P8. **b** Extracted tumor tissues from each treatment group 6 weeks after the DLD-1 xenograft. **c** Inhibition ratios for tumor growth calculated from the mean tumor weights of the control group and the test groups*.* ***P < 0.001. **d** Relative fold changes in the expression of cell cycle regulatory factors between PP* with control and PP*-P8 with control. Each vertical bar represents the arithmetic mean of three replicates. *P < 0.05, **P < 0.01, ***P < 0.001

To determine whether PP*-P8 has anticancer activity in vivo, we assessed its efficacy using the DLD-1 xenograft mouse model. Athymic BALB/c nude mice with subcutaneous DLD-1 xenografts were treated with the commercial chemotherapy drug gemcitabine, PP* or PP*-P8 (see “Methods” for dose and dosage regimen), and the tumor sizes were monitored for 6 weeks before sacrifice (Fig. 2, Additional file 2, Tables S2 and S3). Tumor growth rate was much faster in the untreated control group and the PP*-treated group than in those treated with gemcitabine or PP*-P8 (Fig. 2a). At the end of the experiment, the mean tumor volumes were 2680.9 ± 419.7 mm^3^ in the control group and 2671.1 ± 651.2 mm^3^ in the PP* group, while they were 498.6 ± 192.7 mm^3^ and 1371 ± 349.8 mm^3^ in the gemcitabine and PP*-P8 treatment groups, respectively (Fig. 2a, b; control vs. PP*-P8, *P* = 4.9 × 10^−5^). Tumor weights were 2.13 ± 0.31 g in the control and 2.35 ± 0.32 mm^3^ in PP*, as compared to 0.39 ± 0.16 g in gemcitabine and 0.97 ± 0.30 g in PP*-P8 (Additional file 1, Fig. S2a; control vs. PP*-P8, *P* < 1 × 10^−6^). Inhibition ratios of tumor growth relative to the control were 84.1% and 50.8% in gemcitabine and PP*-P8, respectively (Fig. 2c; control vs. PP*-P8, *P* = 5.3 × 10^−5^). These results demonstrate that our synthetic probiotic PP*-P8 sufficiently suppressed tumor growth similar to that of an anticancer drug.

Next, we asked whether the growth inhibition of the CRC xenograft induced by PP*-P8 is due to cell cycle arrest. Western blot analysis revealed that expression of cell cycle regulatory factors Cyclin B1 and Cdk1 in tumor tissue decreased significantly in response to treatment with PP*-P8 (Fig. 2d, Additional file 1, Fig. S2b). Moreover, expression of p21, which suppresses Cyclin B1/Cdk1, increased after PP*-P8 treatment. In addition, expression of p53 also increased in the PP*-P8-treated group. Overall, the data suggest that the anticancer therapeutic protein P8 inhibits the p53-p21 signaling pathway, resulting in G2 arrest of DLD-1 cells.

### PP*-P8 attenuates tumorigenesis associated with AOM/DSS-induced colitis

**Fig. 3:**
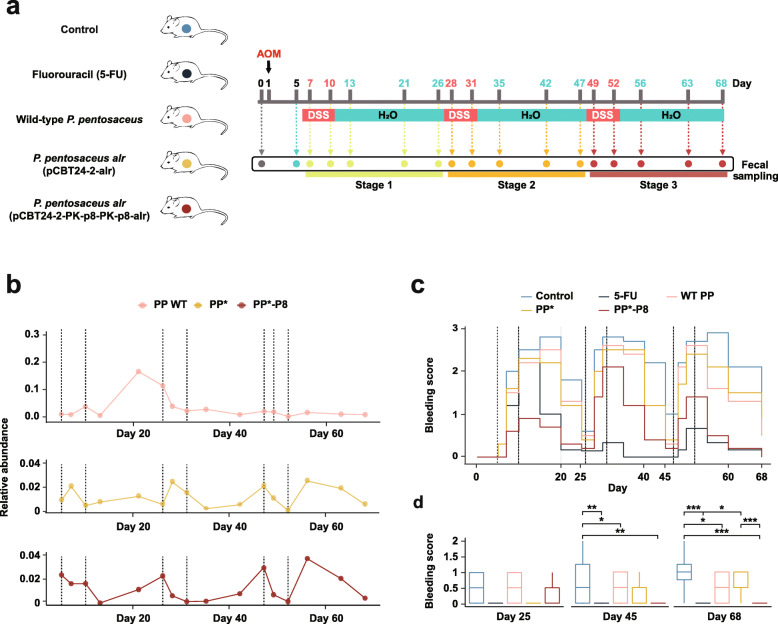
AOM/DSS-induced mouse model of colitis-associated colon carcinogenesis. **a** The experimental scheme for tumor induction by azoxymethane (AOM) and dextran sodium sulfate (DSS). Mice (*n* = 10 in each group) were intraperitoneally injected with 12.5 mg/kg body weight AOM on day 1 and on day 5; they were given water containing 2% w/v DSS for 5 days, followed by regular water for 16 days, which was repeated three times during the 68-day treatment. Treatment groups: untreated control (0.9% saline, oral), fluorouracil (5-FU; 40 mg/kg body weight, intraperitoneal, twice a week), wild-type *P. pentosaceus* (PP WT; 1 × 10^10^ CFU/head, oral, five times a week), *P. pentosaceus alr* (pCBT24-2-alr) (PP*; 1 × 10^10^ CFU/head, oral, five times a week), and *P. pentosaceus alr* (pCBT24-2-PK-p8-PK-p8-alr) (PP*-P8; 1 × 10^10^ CFU/head, oral, five times a week). Schedules for fecal sampling are indicated with arrows. **b** Temporal dynamics of the PP*-P8 population in relative abundance during the experimental period. Dashed lines represent the DSS treatment episodes. **c** Bleeding scores were assessed every 5 days by hemoccult testing and visible signs. Dashed lines represent the DSS treatment episodes. **d** Comparison of bleeding scores in days 25, 45, and 68, the last days of each stage. *P < 0.05, **P < 0.01, ***P < 0.001

We also used the well-established AOM/DSS-inducible murine model for colitis-associated colon carcinogenesis to examine the anticancer effect of the synthetic probiotic PP*-P8 in situ. During the whole experimental period of 68 days, AOM was intraperitoneally injected into C57BL/6 mice on day 1, followed by three episodes of DSS administration in the drinking water. The mice were divided into five groups: untreated control (AOM/DSS only), fluorouracil (5-FU), wild-type *P. pentosaceus* SL4 (PP WT), PP*, and PP*-P8 (Fig. 3a; see “Methods” for dose and dosage regimen). Analysis of the relative abundance of the *Pediococcus* cells in PP WT, PP*, and PP*-P8 indicated that populations were sustained at 0.01~0.03% (Fig. 3b; see “Methods” for microbial community analysis). Although the population of *Pediococcus* in PP WT increased during stage 1, the three groups showed similar relative abundances in the subsequent two stages until the end of the experiment.

**Fig. 4:**
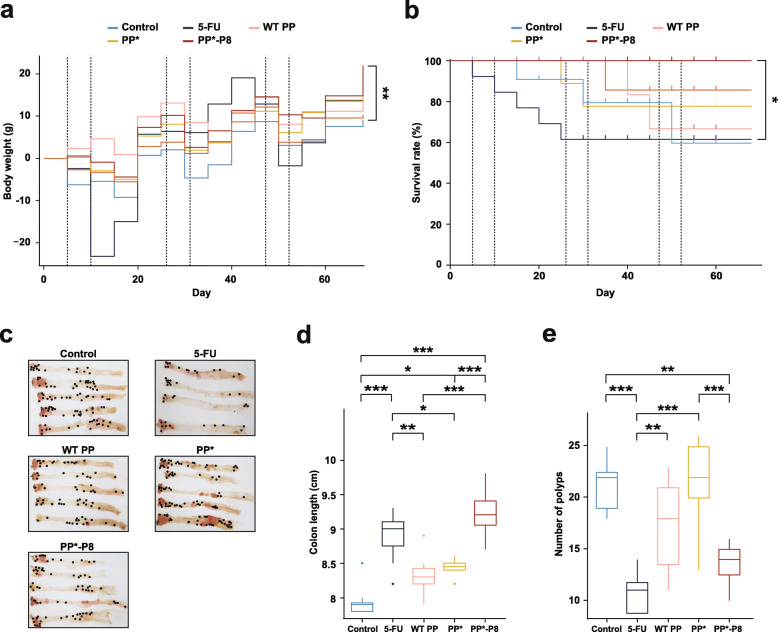
Effects of PP*-P8 on general health and tumorigenesis in the AOM/DSS mouse model. **a** Changes in the bodyweight of mice were recorded each week. **P < 0.01. **b** Kaplan-Meier survival curves for mouse with five different treatment groups. Log-rank test was performed to measure the statistical significance. *P < 0.05. **c** Macroscopic and histopathological appearance of polyps. **d** Colon length and **e** number of polyps were measured after 68 days. *P < 0.05, **P < 0.01, ***P < 0.001

Drastic changes in the average bleeding score were observed before and after each episode of DSS administration (Fig. 3c; *P* = 3.12 × 10^−2^ between day 5 and 10, *P* = 6.40 × 10^−7^ between day 26 and 31, and *P* = 1.90 × 10^−6^ between day 47 and 52). When the bleeding scores of each group were compared on days 25, 45, and 68, which are 14 to 16 days after DSS administration, the PP*-P8 group showed significantly relieved symptoms compared to untreated (*P* = 1.32 × 10^−2^) on day 45, untreated (*P* = 1.00 × 10^−5^) and PP* (*P* = 3.53 × 10^−4^) on day 68 (Fig. 3d, Additional file 2, Tables S4 and S5). Severe bleeding and bleeding around the anus were often noticeable in the controls, whereas only occult blood or slight bleeding was detected for PP*-P8. Figure 4a as well as Tables S4 and S5 in Additional file 2 showed that DSS treatment had negative effects on weight gain in the 5-FU and control groups, while bodyweight of mice in the PP*-P8 group increased until the end of the experiment. Kaplan-Meier survival plot similarly showed that, with no fatalities, PP*-P8 treatment increased the survival of AOM/DSS-treated mice during the experiment (Fig. 4b). Notably, survival rate of the 5-FU group was extensively reduced during stage 1 and significantly different from PP*-P8 (*P* = 0.025). Colon length is one of the markers for evaluating colonic inflammation severity and was measured after animals were euthanized to reveal that the three control groups had significantly decreased colon lengths in comparison to PP*-P8 (*P* < 1 × 10^−6^, *P* = 2 × 10^−6^, *P* = 1.8 × 10^−5^ for untreated, PP WT, PP*, respectively), which was indicative of severe inflammation (Fig. 4c, d). In comparison to the colon length of untreated control, which was administered with AOM/DSS only, the colon length of PP*-P8 was close to that of the healthy mouse group, indicating that PP*-P8 treatment prevents the colon from being shortened due to the presence of AOM/DSS (Additional file 2, Table S4).

The number of nodular polypoid tumors located in the middle and distal colon in the PP*-P8 treatment group was lower than those in untreated control (*P* = 3.08 × 10^−3^) and PP* (*P* = 1.68 × 10^−3^) groups, while there was no significant change in PP WT (*P* = 0.3) (Fig. 4e). We also tested the effect of lyophilized PP*-P8 resuspended at 1/10 of the original culture volume on anticancer activity in the AOM/DSS mouse model; there was no significant difference in the number of polyps between the original PP*-P8 and the lyophilized (Additional file 1, Fig. S3). Taken together, these results from the AOM/DSS-induced colitis-associated cancer model indicate that the orally administered PP*-P8 probiotic effectively inhibited inflammation-associated carcinogenesis and tumor development in the colon.

### PP*-P8 modulates gut microbiota to alleviate AOM/DSS-induced dysbiosis

We further explored the possible impacts of the synthetic probiotic PP*-P8 on gut microbiota in the AOM/DSS murine model for colitis-associated colon cancer. C57BL/6 mice were subjected to a dose regimen and a fecal sampling schedule that was divided into untreated control, 5-FU, PP WT, PP*, and PP*-P8 treatment groups (Fig. 3a). Using DNA from the fecal samples, amplicon sequencing of the V3–V4 region of the 16S ribosomal RNA gene was performed to monitor microbial community structure. Processed reads were clustered into amplicon sequence variants (ASVs) with a 99% threshold for sequence identity using QIIME 2 [20] to calculate relative abundance (Additional file 2, Tables S6 and S7).

**Fig. 5:**
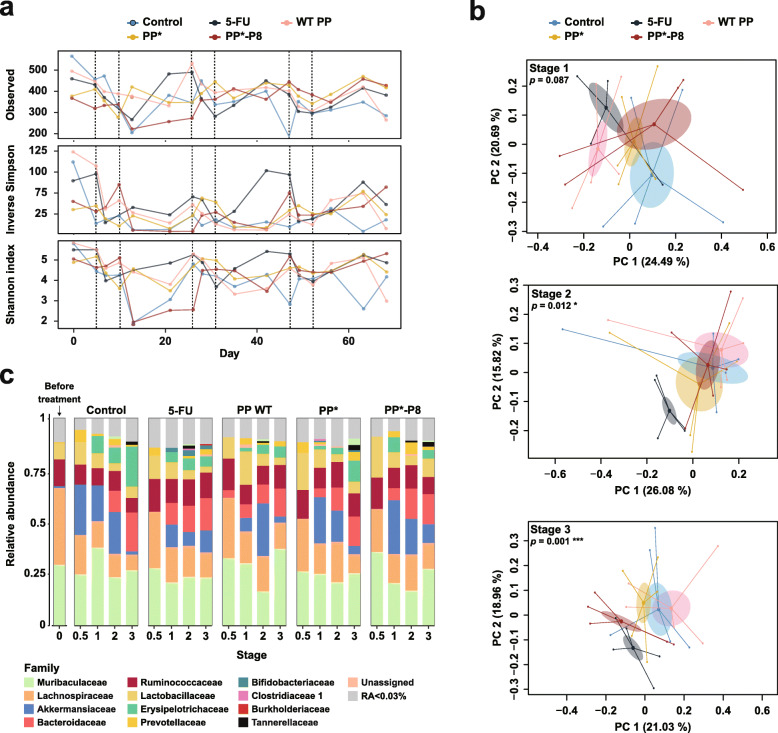
Longitudinal analyses of the gut microbiota of AOM/DSS mice treated with PP*-P8. **a** Changes in alpha diversity indices of microbial communities in the fecal samples. Species richness and evenness are plotted as the number of observed ASVs and inverse Simpson and Shannon indices. **b** Principal coordinate analysis based on Bray-Curtis dissimilarity. Each dot indicates a single sample and each group is shown in a different color. *P* values correspond to the permutational multivariate analysis of variance results. **c** Microbial composition at the family level is shown as relative abundance. Except for stage 0.5, which shows a single sample, proportions are the averages of five samples. D0, day 0

Species richness and evenness were measured by the number of ASVs and the inverse Simpson index, respectively, to evaluate microbial diversity, which was severely disturbed by AOM/DSS treatment (Fig. 5a). As expected, all the experimental groups lost alpha diversity, which reduced the number of ASVs during each DSS administration; however, the ASVs partially recovered until the next administration. Interestingly, the PP*-P8 group seemed to restore taxonomic diversity in stage 3 better than the 5-FU and control groups toward the end of the experiment (red lines in Fig. 5a). Principal coordinates analysis (PCoA) based on Bray-Curtis dissimilarity [21] illustrated the dissimilarities of fecal microbiota between each treatment group and pre-treated samples on day 0 and day 5 increased as stages of treatment progressed (Additional file 1, Fig. S4). The differences between the controls, 5-FU, and PP*-P8 treatments were not obvious during stage 1 (Fig. 5b, Additional file 1, Fig. S4); however, beta diversity increased over time and permutational multivariate analysis of microbial variance resulted in significant statistical differences among the groups in stages 2 and 3 (*P* = 0.012 and *P* = 0.001, respectively). The PCoA plots also show that the three control groups became more dispersed in stages 2 and 3 than PP*-P8. It is noteworthy that 5-FU and PP*-P8 appeared similar in stage 3 (bottom panel of Fig. 5b).

Distribution and abundance of microbial taxa for each group in each stage were examined and the results indicated that bacteria in the *Bacteroidetes* and *Firmicutes* phyla dominated the mouse gut microbiota (Additional file 2, Table S7). Relative abundance at the family level illustrated that on day 0 *Muribaculaceae*, *Lachnospiraceae*, *Ruminococcaceae*, and *Lactobacillaceae* were the main families, while during DSS administration *Akkermansiaceae*, *Bacteroidaceae*, and *Erysipelotrichaceae*, as well as *Muribaculaceae*, *Lachnospiraceae*, *Lactobacillaceae*, and *Ruminococcaceae* were the primary bacteria (Fig. 5c). The relative abundance of each family fluctuated and depended on the stage of the DSS treatment. Compared to the control, the most different beta diversity pattern was observed at stage 3 (Fig. 5b; *P* = 0.001), and the 5-FU-treated group was enriched with *Akkermansiaceae* and *Ruminococcaceae* but depleted of *Erysipelotrichaceae* and *Lactobacillaceae*. In the PP*-P8 treatment group, *Akkermansiaceae* and *Lactobacillaceae* increased, while *Erysipelotrichaceae* decreased compared to the controls. Our data from the AOM/DSS mouse model demonstrate that the PP*-P8 probiotic contributes to alleviating dysbiosis induced by AOM/DSS by modulating gut microbiota structure with respect to alpha and beta diversity and the proportion of potentially beneficial taxa.

### Specific bacterial taxa are associated with eubiosis maintained by PP*-P8

**Fig. 6:**
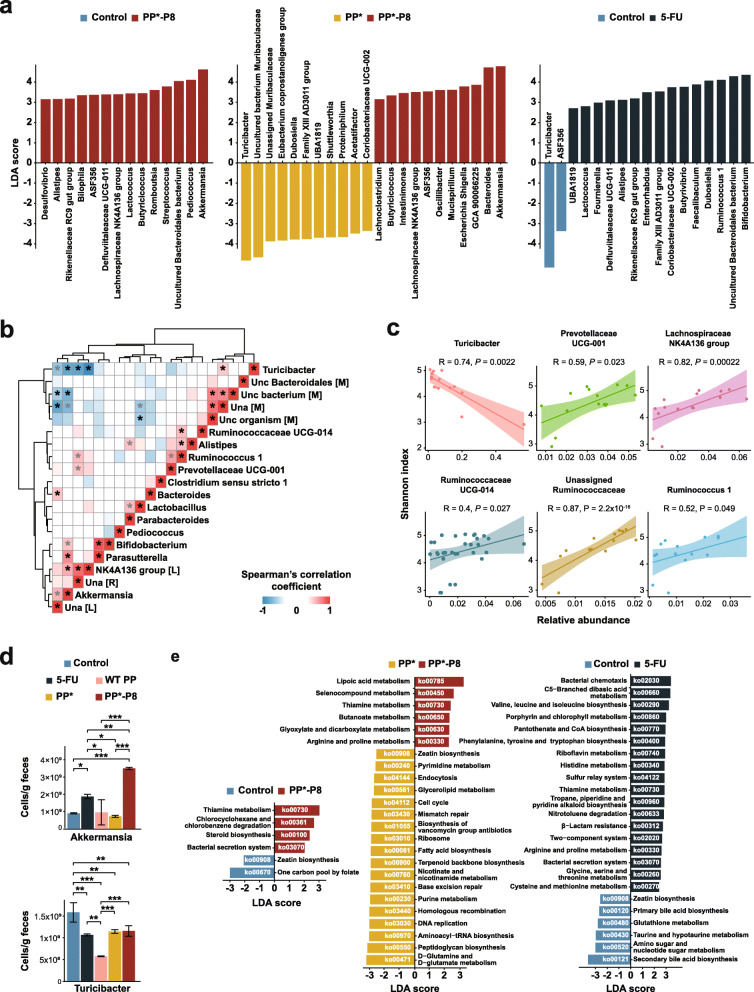
Specific microbial taxa likely associated with differences between the treatment groups. **a** Linear discriminant analysis effect size of samples after the final DSS administration. **b** Positive and negative correlation matrix between the top 20 most abundant bacterial taxa. Results of a pairwise Spearman’s rank correlation after the final DSS administration are shown. Correlations with *P* values less than 0.05 are marked with asterisk symbols and adjusted *P* values less than 0.05 by the Benjamini-Hochberg FDR method are colored black. Related genera based on Euclidean distance were clustered together. Red, positive correlation; blue, negative correlation. Uncultured (unc) or unassigned (una) genera were labeled with the initials of their family names, [M], [L], and [R] represent *Muribaculaceae*, *Lachnospiraceae*, and *Ruminococcaceae*, respectively. **c** Scatterplot with Spearman’s rank correlations between the relative abundances of six genera and Shannon indices. Those with significant *P* values in **b** are shown in **c**. **d** Quantification of the number of cells for *Akkermansia* and *Turicibacter* as measured by quantitative PCR in fecal samples on day 68 after the final DSS treatment. *P < 0.05, **P < 0.01, ***P < 0.001. **e** Discriminative functional pathway abundant between control versus PP*-P8, PP* versus PP*-P8, and control versus 5-FU

To determine which bacteria are most likely responsible for the differences between the treatment groups, we applied the linear discriminant analysis (LDA) and effect size (LEfSe) [22] method to calculate the LDA scores for the last three samples on days 56, 63, and 68 in stage 3 when the mice are recovering from the last DSS administration. The lists of taxonomic clades at the genus level, ranked according to the effect size, that are differential among groups with statistical and biological significance are shown in Fig. 6a. They indicated that, between the PP*-P8 and control groups, *Akkermansia*, *Pediococcus*, and an uncultured *Bacteroidales* bacterium were most discriminative (log_10_ LDA ≥ 4.0) in PP*-P8. Similarly, *Akkermansia* and *Bacteroides* were most differential in PP*-P8, whereas *Turicibacter* and an uncultured *Muribaculaceae* bacterium were in PP*. Between 5-FU and control, most discriminative in 5-FU included, *Bifidobacterium*, an uncultured *Bacteroidales* bacterium*, Ruminococcus* 1, and *Dubosiella*, while one in control was *Turicibacter*. Between PP*-P8 and 5-FU, *Bacilli* at various taxonomic ranks down to *Lactobacillus*, *Pediococcus*, and other lactobacilli were most distinctive in PP*-P8, and *Actinobacteria* at various ranks down to *Bifidobacterium*, *Coriobacteriia* down to *Coriobacteriaceae* UCG-002, and *Dubosiella* were in 5-FU (Additional file 1, Fig. S5).

Interactions between members of gut microbiota were identified using a pairwise Spearman’s rank correlation coefficient, which was calculated for days 56, 63, and 68 in stage 3 and visualized as a heat map for systematic analysis using 20 most abundant genera (Fig. 6b). The plot revealed two competing clusters: one composed of *Akkermansia*, an unassigned *Lachnospiraceae*, the *Lachnospiraceae* NK4A136 group, an unassigned *Ruminococcaceae*, *Bifidobacterium*, and *Parasutterella*, and the other *Turicibacter*, an uncultured *Bacteroidales* bacterium*,* unassigned or uncultured members of *Muribaculaceae*. Specific bacteria such as *Akkermansia* and an unassigned *Lachnospiraceae* as well as *Turicibacter* and an unassigned or an uncultured member of *Muribaculaceae* have multiple interactions with other bacteria and, thus, can be called keystone taxa. *Akkermansia*, the signature taxon of the PP*-P8 group’s microbial profile, had highly negative correlations with *Turicibacter*, which is a biomarker for control and PP*, an uncultured member of *Muribaculaceae*, and an unassigned *Muribaculaceae*. On the other hand, *Akkermansia* had strong positive correlations with *Lachnospiraceae* NK4A136, *Parasutterella, Bifidobacterium*, and an unassigned *Lachnospiraceae*. *Turicibacter* had negative correlations with *Lachnospiraceae* NK4A136, an unassigned *Ruminococcaceae, Akkermansia*, and an unassigned *Lachnospiraceae* and positive correlations with an unassigned *Muribaculaceae*. These results suggest that positive or negative relationships among microbial members shape the community structure.

We then explored the possible implication of specific bacterial taxa in alpha diversity. Associations of the relative abundances of 20 most abundant genera with Shannon indices were measured with Spearman’s rank correlation (Fig. 6c). *Turicibacter* was the only genus that showed a significant negative correlation with the Shannon index (*R* = − 0.74, *P* = 0.0022). Conversely, *Lachnospiraceae* NK4A136 (*R* = 0.82, *P* = 0.00022) and an unassigned *Ruminococcaceae* (*R* = 0.87, *P* = 2.2 × 10^−16^) had positive correlations. Based on these, we speculate that the cluster that consists of *Turicibacter* and some members of *Muribaculaceae* is associated with dysbiosis, while one that includes *Akkermansia*, some members of *Lachnospiraceae*, an unassigned *Ruminococcaceae*, *Bifidobacterium*, and *Parasutterella* is associated with eubiosis.

Based on amplicon sequencing, the average relative abundance of *Akkermansia* in PP*-P8 during days 56, 63, and 68 of recovery in stage 3 was 0.09 ± 0.02%, which is 4.16- and 2.77-folds higher than PP* and the control, respectively. On the other hand, those of *Turicibacter* in the control and PP* during those days were 0.27 ± 0.2% and 0.11 ± 0.03%, respectively. They are 8.89- and 3.88-folds higher than PP*-P8, respectively, and 9.39- and 4.1-folds higher than 5-FU, respectively. To validate the outcomes of proportion-based analyses, we determined absolute abundances of the two genera using quantitative real-time polymerase chain reaction (Fig. 6d, Additional file 2, Table S4). *Akkermansia* in PP*-P8 was measured at 4.21 ± 0.82 × 10^9^, 1.17 ± 0.07 × 10^9^, and 3.51 ± 0.06 × 10^9^ cells/g feces in days 56, 63, and 68, which are 3.5~4.9-fold higher than those in PP* and 2.74~3.93-fold higher than the control. In the case of *Turicibacter*, 1.27 ± 0.02 × 10^9^, 2.08 ± 0.61 × 10^9^, and 1.59 ± 0.22 × 10^9^ cells/g feces were measured in the control and 1.75 ± 0.03 × 10^9^, 1.46 ± 0.03 × 10^9^, and 1.14 ± 0.04 × 10^9^ cells/g feces in PP* during those days. They are 0.99~3- and 0.98~2.11-folds higher than PP*-P8, respectively, and 1.45~2.43- and 1.07~1.99-folds higher than 5-FU, respectively. These results demonstrate that the relative abundances of both *Akkermansia* and *Turicibacter* are consistent with quantitative enumeration.

To identify the functional features of each group during days 56, 63, and 68 in stage 3, significantly enriched KEGG pathways predicted by PICRUSt2 were listed according to the LDA effect size (log_10_ LDA > 2.0) (Fig. 6e). Microbial members in PP*-P8 as compared to the control contributed to thiamine metabolism (ko00730), chlorocyclohexane and chlorobenzene degradation (ko00361), steroid biosynthesis (ko00100), and bacterial secretion system (ko03070). Between PP*-P8 and PP*, abundant pathways in PP*-P8 included cofactor and vitamin metabolism (ko00785, ko00730), carbohydrate metabolism (ko00630, ko00650), and selenocompound and arginine/proline metabolism (ko00450, ko00330). Those in PP* included d-glutamine and d-glutamate metabolism (ko00471), peptidoglycan biosynthesis (ko00550), protein biosynthesis (ko00970, ko03010), and pathways involved in replication and repair (ko03030, ko03440, ko03410, ko03430). In both comparisons, thiamine metabolism was in common. Eighteen pathways were predicted from 5-FU as significant functional profiles compared to control: bacterial chemotaxis (ko02030), C5-branched dibasic acid metabolism (ko00660), six pathways involved in amino acid metabolism (ko00290, ko00400, ko00340, ko00330, ko00260, ko00270), and four pathways for cofactor and vitamin metabolism (ko00860, ko00770, ko00740, ko00730). Notably, PP*-P8 and 5-FU had thiamine metabolism in common. Also, zeatin biosynthesis (ko00908) was repeatedly detected in the control and PP*.

## Discussion

Advances in the mechanistic understanding as well as diagnosis and treatment of cancer have increased the success rate of recovery, but there remain concerns about the side effects and drug resistance associated with current treatment programs. Among biopharmaceutical approaches to cancer treatment, efforts to apply live bacteria as therapeutic agents were verified in preclinical or early clinical trials; although they still have toxicity issues, these are genetically attenuated to less virulent or toxin-free levels [23–25].

Similar to that reported in our work, commensal bacteria recently received substantial attention from their potential for suppressing or preventing CRC [26–29]. Previously, we discovered a novel therapeutic peptide originating from a probiotic LAB strain and confirmed its clinical potential for the anti-CRC efficacy using a recombinant form [11]. In the present study, we established a stable and efficient DDS by adopting a d-Ala auxotrophic mutant of the food-grade LAB *P. pentosaceus* SL4(-7) complemented with an *alr*-containing plasmid expressing dual gene cassettes under the control of the PK-PK promotor system. Each of these cassettes encodes a signal peptide for secretion that can be fused with a therapeutic protein. We then loaded the bacterium with the novel therapeutic protein P8 from *L. rhamnosus* CBT LR5 that has a strong anti-proliferative activity against DLD-1 cells [11, 30], to engineer the PP*-P8 synthetic probiotic for CRC therapy. Its efficacy was validated by two different murine models, DLD-1 xenograft and AOM/DSS-induced CRC. The xenograft model showed that our synthetic probiotic effectively inhibits tumor growth and can be a competitive therapeutic strain. The AOM/DSS model was used to longitudinally evaluate the inhibitory effects of our synthetic probiotic on carcinogenesis and demonstrated normal body weight and colon length, as well as a reduced fatality, bleeding score, and the number of polyps, in the PP*-P8-treated mice as compared to controls.

Gemcitabine, a nucleoside analog, has been widely used as a standard anticancer drug for solid tumors [31] and applied to patient-derived tumor engraftment models with non-small cell lung cancer or pancreatic cancer [31–33]. However, it is rapidly inactivated by cytidine deaminase [31] or deoxycytidylate deaminase [34] before being delivered to the target site, and thus inappropriate for systemic therapy. Consequently, we treated gemcitabine, generally prescribed for solid tumors, by intraperitoneal injection in the DLD-1 xenograft model and used 5-FU, the first-line regimen for colorectal cancer [34, 35], in the AOM/DSS model for systemic chemotherapy administration.

There is an increasing awareness of the roles of the gut microbiome in influencing the response to and outcome of chemotherapy [19, 36, 37]. Reciprocal modification of the microbiota by chemotherapeutic agents is also increasingly appreciated. One important observation from our AOM/DSS experiment is that the PP*-P8 probiotic modulates the gut microbiota structure to alleviate the change from eubiosis to dysbiosis induced by AOM/DSS. Loss of diversity, increase in perturbation, imbalance in composition, and changes to specific lineages, either beneficial or deleterious, are hallmarks of the unhealthy status of microbiota in the gut [38]. Recovery of alpha diversity and coherence of the microbial communities after three DSS treatments were most prominent in the PP*-P8 group, suggesting that our synthetic probiotic is not only effective in treating CRC but also helpful in maintaining the microbiota structure and possibly securing host health benefits. Increased body weight, survivability, and colon length measured in PP*-P8-treated mice support this hypothesis [39]. Apart from the anticancer effect, rapid weight loss, decreased survivability, and shortening of colon length in the 5-FU-treated group were consistent with previously studied cases and are indicative of the double-sidedness of chemotherapy [40–44].

Results from LEfSe between groups enlisted specific microbial taxa that are discriminative with statistical and biological significance during PP*-P8 treatment in the AOM/DSS model. Among the taxa identified, the most notable was the *Akkermansia*-*Verrucomicrobia* clade. *Akkermansia muciniphila* is a well-known biomarker for defining the healthy gut microbiota and is considered a promising candidate for next-generation probiotics [45–47]. In accordance with our study, several studies recently reported this species’ attenuation effects on AOM/DSS-induced inflammation [48, 49] and other clinical parameters [50]. Conversely, *Turicibacter* was most characteristic of the untreated control and PP*. Although little is known about this genus, increasing reports about their association with disease solidifies our results. The type strain of *Turicibacter sanguinis* was isolated from the blood culture of a febrile patient with acute appendicitis [51]; moreover, some *Turicibacter* bacteria are reported to have a pathobiont lifestyle [52] and are often relevant to host inflammation [53–55]. Also, *Turicibacter* was abundantly detected in tumor-bearing mice treated with AOM/DSS [56] or significantly enriched in Japanese subjects who suffer from constipation [57]. Based on Spearman’s correlation analysis, *Akkermansia* and *Turicibacter* were identified as keystone taxa because they seem to shape antagonistic clusters, consisting of members from LEfSe results and Shannon diversity correlations. We, therefore, hypothesize that the PP*-P8 probiotic coordinates the microbial consortium to maintain eubiosis during AOM/DSS-induced colitis-associated carcinogenesis and likely helps improve drug response and reduce relapse rate. Future work is warranted to investigate these hypotheses.

It is interesting that the microbial communities of mice treated with PP*-P8 or 5-FU [34] were similar at stage 3 in the AOM/DSS model, and both treatment groups presented high *Akkermansia* and low *Turicibacter* populations. With the exception of their effects on cancer development inhibition, it is unclear how these two treatments fundamentally different in nature affect the microbiota structure to resemble each other. It seems though that they influence the microbiota through different mechanisms of action, either directly or indirectly. Indeed, other than *Akkermansia*, lactobacilli including the treated *Pediococcus* were distinctive in PP*-P8, while bifidobacteria were in 5-FU. It should be cautioned, however, to conclude that 5-FU administration during AOM/DSS treatment has health benefits because the molecule itself causes various side effects that include DNA damage, inflammation [34, 58–61], and decreased survivability as observed in our study.

## Conclusions

Our approach to treating CRC with a stable and effective synthetic probiotic presents the validity and feasibility of cell-based designer biopharmaceutical agents. Our results also bear testimony to the positive or negative influences of biopharmaceuticals as well as chemotherapeutics on gut microbiota and possibly general health. Considering their potential impact, we suggest scrutinizing the dynamics of the microbiome and associated health issues during development of pharmaceuticals that are targeted to treat or prevent cancer, including CRC.

## Methods

### Bacterial strains and culture

The anticancer protein P8 was identified from *Lactobacillus rhamnosus* CBT LR5 (= KCTC 12202BP) isolated from the human intestine. *P. pentosaceus* SL4(-7) that was used as a drug delivery vehicle is a derivative of *P. pentosaceus* SL4 (= KCTC 10297BP) isolated from the traditional Korean fermented vegetable kimchi. These strains were derived from the culture collection of Cell Biotech Co., Ltd., Gimpo, Korea, and routinely statically cultured at 37 °C for 18–24 h in Man, Rogosa and Sharpe broth (Difco, Detroit, MI, USA) or M9 broth with 1% glucose for protein expression. *Escherichia coli* DH5α was cultured for 18–24 h in Lysogeny broth (Difco) at 37 °C. Under the strictly anaerobic condition, *A. muciniphila* KCTC 15667 was statically cultured for 48 h in brain heart infusion broth (Becton, Dickinson and Company, Bergen County, NJ, USA) with 3% of mucin from the porcine stomach (Sigma-Aldrich, St. Louis, MO, USA) at 37 °C. *T. sanguinis* DSM 14220 was strictly anaerobically cultured for 48 h in chopped meat broth (Becton, Dickinson and Company, Bergen County, NJ, USA) at 37 °C.

### Cell culture

The human CRC cell line DLD-1 was purchased from the Korean Cell Line Bank and maintained under 5% CO_2_ and 37 °C in Roswell Park Memorial Institute (RPMI)-1640 medium (Gibco, Grand Island, NY, USA) containing 10% fetal bovine serum (Gibco) and 1% penicillin/streptomycin (Gibco).

### Construction of a plasmid-encoded *alr* complementation system

We followed the genetic design of the d-Ala auxotrophic PP as previously described [62]. To generate DNA fragments flanking the *alr* gene, we synthesized the regions Hr1 and Hr2 1-kb upstream and downstream of *alr* and the Amp-resistant gene in between Hr1-Amp^R^-Hr2, and then cloned it into pCBT24-2 (KCCM12182P). The in-frame deletion of *alr* was made by homologous recombination with a pCBT24-2-alrHr1,2-Amp^R^ construct. After electroporation (1.24 kV, 25 μF, 1-mm cuvette), among of PP transformants, the d-Ala auxotrophic PP was selected using MRS agar with 10 μg/ml erythromycin. The in-frame deletion mutants (*Δalr*) were screened on MRS agar containing erythromycin and 200 μg/ml d-Ala, and then the selected mutant was verified by PCR using the primers shown in Table S1 in Additional file 1. The PCR product was sequenced and verified. Selected mutants were complemented with the pCBT24-2-alr plasmid for the *alr* auxotroph complementation system *P. pentosaceus alr* (pCBT24-2-alr), PP*.

### Construction of the P8 dual-promoter gene expression systems

Two-promoter systems were introduced for maximal production of P8 in PP*. Usp45-P8 fragments were fused with five pairs of two promoter sets (Cosmo Genetech Co., Ltd., Seoul, Korea): PK-Usp45-p8-PK-Usp45-p8, PK-Usp45-P8-ChoS-Usp45-p8, GK-Usp45-p8-PK-Usp45-p8, GK-Usp45-p8-GK-Usp45-p8, and GK-Usp45-p8-LDH-Usp45-p8. Each expression system was inserted into the pCBT24-2-alr plasmid using *Nhe*I/*Sal*I and *BamH*I/*Pst*I restriction enzymes and transformed into the *alr* knockout mutant. Finally, the pCBT24-2-PK-p8-PK-p8-alr plasmid (accession number KCCM12181P) was selected as DDS for P8 (Additional file 1, Fig. S1a).

### ELISA analysis of P8 concentration

A 96-well polystyrene plate (SPL Life Sciences; Pocheon-si, Gyeonggi-do, Korea) was coated with 100 μl diluted anti-P8 IgG (1:5500 poly clonal-rabbit; Young In Frontier Co., Ltd., Seoul, Korea) in ELISA coating buffer (Bethyl Laboratories; Montgomery, TX, USA) overnight at 4 °C. After coating, the wells were washed twice with 300 μl wash buffer (1× Tris-Buffered-Saline Buffer (TBS) with 0.05% Tween 20 (TBS-T)) followed by blocking with 300 μl blocking buffer (1× phosphate-buffered saline (PBS) and 5% fetal bovine serum (FBS; Gibco) for 1 h at room temperature (RT). The wells were washed three times with 300 μl wash buffer prior to adding 100-μl protein samples (culture supernatant or mouse serum), followed by a 150-min incubation at RT. After sample binding, the wells were washed four times with 300 μl wash buffer (TBS-T) followed by primary antibody binding with 100 μl biotinylated anti-P8 IgG (500 pg/ml anti-P8 IgG-biotin; Young In Frontier Co., Ltd.) in 1× PBS with 5% FBS followed by a 90-min incubation at RT. After primary antibody binding, the wells were washed four times with 300 μl wash buffer (TBS-T), followed by secondary antibody binding with 100 μl streptavidin-HRP (166 pg/ml Young In Frontier Co., Ltd.) in 1× PBS with 2.5% FBS and incubated for 30 min at RT. After secondary antibody binding, the wells were washed four times with 300 μl wash buffer (TBS-T) followed by color development with 100 μl TMB one solution (Bethyl Laboratories) for 20 min at RT under dark and then 50 μl stop buffer (Bethyl Laboratories). Absorbance was measured using the multifunctional microplate reader (SpectraMax M5; Molecular Devices, Sunnyvale, CA, USA). A standard curve for the recombinant P8 sera dilution (2-fold dilutions 1000 pg/ml to 15.625 pg/ml) was performed in triplicate. Each sample was assayed in two different dilutions and run in duplicate. Results are reported in picogram amounts per milliliter PP*-P8 protein.

### Mouse strains and growth conditions

Male athymic nude mice (BALB/cAnN.Cg-Foxn1nu/CrlNarl; 5 weeks of age, 50 in total for the CRC xenograft model) and male C57bL-6J mice (C57bL-6J; 8 weeks of age, 50 in total for AOM/DSS-induced CRC model) were purchased from SR Bio (Gyeonggi-Do, Korea). Mice were housed at the constant temperature (20 ± 3 °C) and humidity (40 ± 20%) conditions with a 12/12-h light/dark cycle in a specific pathogen-free facility (Laboratory Animal Center of Cell Biotech Co., Ltd., Korea). The animals had free access to irradiation-sterilized dry pellet-type feeds and water during the study period. In accordance with the study schedule, the mice were sacrificed by CO_2_ inhalation at the end of the experiment. All animal experimental protocols were reviewed and approved by the Institutional Animal Care and Use Committee board in the Cell Biotech (IACUC, approval no. study I, CBT-2018-02; study II, CBT-2018-03) based on guidance of the Association for Assessment and Accreditation of Laboratory Animal Care (AAALAC).

### CRC xenograft mouse model

A xenograft mouse model for CRC was developed using human-derived DLD-1 cells. DLD-1 cells were inoculated in RPMI1640 (Gibco) medium supplemented with 10% FBS (Gibco) and 0.1 mM NEAA (Gibco). At the exponential growth phase, DLD-1 cells were harvested and counted for tumor inoculation. For tumor development, 2 × 10^6^ DLD-1 tumor cells were suspended in 0.1 ml PBS and used to subcutaneously inoculate the rear right flank of each mouse. Seven days after tumor inoculation, the animals were weighed and measured for tumor volume and then randomly divided into five groups with seven animals each using a randomized block design for homogeneous group formation when the mean tumor size reached approximately 100–150 mm^3^ (5 days). Tumor volumes were measured using the following formula: volume = (width/2)^2^ × length, where length and width represent the largest and shortest tumor diameters, respectively. Mice were euthanized when tumor volume reached approximately 3000 mm^3^. This end-point tumor size was chosen to maximize the number of tumor doublings within the exponential growth phase in the untreated group. Inhibition ratios were determined by IR (%) = (1 − *T*/C) × 100 where *T* is the mean tumor weight of the test substance and *C* is the mean tumor weight of the negative control group.

### AOM/DSS-induced CRC mouse model

For the AOM/DSS-induced CRC model, cohoused age- and sex-matched 6-week-old mice were intraperitoneally injected with AOM (Sigma-Aldrich) with 12.5 mg/kg body weight on the first day of experiment. After 5 days, mice were treated with 2% (wt/vol) DSS (molecular weight 36–50 kDa; MP Biomedicals, Irvine, CA, USA) in their drinking water for 5 days, followed by 16 days of regular water. This cycle was repeated three times.

The presence of occult blood (or gross blood) in the rectum and body weight were determined every 5 days each week for each mouse. Bleeding analysis was scored as 0 when there was no blood in the hemoccult test, 1 for a positive hemoccult result, 2 for slight bleeding, and 3 for gross bleeding and bleeding around the anus. Weight changes during the experiment were calculated as the percent change in weight compared with the baseline measurement. Survival curves were drawn using the Kaplan-Meier method in Prism (version 8.0.2, Graph Pad Software, Inc.). Mice were sacrificed on day 68 and histopathological examination was assessed to measure colon length and number of polyps.

### Administration of anticancer drugs

For the CRC xenograft model, mice were randomly divided into different treatment groups when their average body weight reached 22 ± 2 g. Average mean tumor sizes were 100–1500 mm^3^ (*n* = 10) 7 days post tumor inoculation. Oral administration started with 0.9% saline (control), 60 mg/kg gemcitabine, 1 × 10^10^ CFU/head PP*, and 1 × 10^10^ CFU/head of PP*-P8. The treatment was administered five times each week for 6 weeks. As a positive control, 60 mg/kg gemcitabine were intraperitoneally injected twice a week. For the AOM/DDS induced CRC model, mice were randomly divided into treatment groups (*n* = 10) when the average body weight reached to 22 ± 2 g. To test anticancer activity of the synthetic probiotics, 0.9% saline (control), 1 × 10^10^ CFU/head PP*, 1 × 10^10^ CFU/head of PP*-P8, 1 × 10^11^ CFU/head of lyophilized PP*-P8-L, and 1 × 10^10^ CFU/head of PP WT were orally administrated to each group five times a week for 68 days. 40 mg/kg of 5-FU was intraperitoneally injected twice a week as positive controls.

### Western blot

PP*-P8 on MRS agar plates were used to inoculate 10 ml MRS broth containing 10 μg/ml erythromycin and cultured at 37 °C for 15 h without shaking. One milliliter of pre-culture was used to inoculate 10 ml modified M9 medium containing 10 μg/ml erythromycin and cultured at 37 °C for 48 h without shaking. Next, 5 ml of the culture was centrifuged, and the supernatant was collected. The supernatant was concentrated using the TCA precipitation method (25% TCA, −20 °C, 1 h) to isolate total protein. Finally, the P8 protein was detected by western blotting.

To extract total protein from mouse xenograft tissues (DLD-1-derived), the ground tissue powder was lysed in RIPA buffer containing a protease inhibitor cocktail (Roche). Proteins samples were separated by sodium dodecyl sulfate polyacrylamide gel electrophoresis and transferred to a polyvinylidene difluoride membrane (Amersham Bioscience, Piscataway, NJ, USA). Blotted membranes were blocked in 5% skimmed milk/TBS-T and incubated overnight at 4 °C with the appropriate primary antibodies (rabbit anti-P8 antibody, Young In Frontier Co., Ltd; Seoul, Korea; commercial p53, p21, Cdk1, cyclin B1, and glyceraldehyde 3-phosphate dehydrogenase (GAPDH) antibodies, Cell Signaling Technology, Danvers, MA, USA) diluted 1:1000. The membranes were washed for 15 min three times with TBS-T and then blocked in 5% skimmed milk/TBS-T. The membranes were then incubated for 1 h at 4 °C with an HRP-linked secondary antibody (Cell Signaling Technology). GAPDH was used as an internal control. Protein bands were detected using an enhanced chemiluminescence kit (Millipore, Billerica, MA, USA) followed by autoradiography with a Chemi-doc™ Touch Imaging System (Bio-Rad Laboratories, Hercules, CA, USA).

### DNA extraction and sequencing

Fecal samples were aseptically collected and frozen at −80 °C throughout the experimental period. After the final sampling, all samples were thawed slowly and measured into 200 mg aliquots. DNA was extracted using a Fast DNA SPIN kit for fecal samples (MP Biomedicals) according to the manufacturer protocol. Extracted DNA was further processed on an Illumina platform by an external service (Macrogen, Seoul, Korea). The V3-V4 region of the 16S ribosomal RNA gene was targeted for amplicon sequencing using sequence-specific primers (337F: CCTACGGGA(N)GGCWGCAG, 806R: GACTACHVGGGTM(A)TCTAAT) with attached Illumina adapter overhang sequences (Forward: CGTCGGCAGCGTCAGATGTGTATAAGAGACAGCCTACGGGNGGCWGCAG, Reverse: GTCTCGTGGGCTCGGAGATGTGTATAAGAGACAGGACTACHVGGGTATCTAATCC). For library construction, the Herculase II Fusion DNA polymerase Nextera XT index Kit v2 was used and sequenced using the Illumina MiSeq platform.

### Bioinformatic analysis of microbial communities

Sequence analysis was performed using QIIME 2 [20] (version 2019.10). Raw sequences were demultiplexed and denoised with DADA2, and a feature table was generated (Additional file 2, Tables S6 and S7). The MAFFT [63] and FastTree [64] programs were used via the q2-phylogeny plugin to perform a multiple sequence alignment of the representative sequences and construct a phylogeny tree. Taxonomic assignment of ASVs was conducted by q2-feature-classifier plugin based on the SILVA 16S rRNA gene database. The feature table and phylogenetic distances were imported into R Studio (version 1.1.383, R Studio, Inc., Boston, MA, USA) for downstream analysis. The feature table was rarefied by random subsampling without replacement to stimulate even number of reads per sample. Alpha diversity, including richness, and inverse Simpson and Shannon indices were measured with the phyloseq (version 1.30.0) and vegan (version 2.5-6) packages in R. PCoA plot was generated with Bray-Curtis distance and permutational multivariate analysis of variance (PERMANOVA) tests using 999 permutations to evaluate group dissimilarity using the Adonis function in the vegan R package. To determine the discriminative genera between groups, LDA scores were calculated by LEfSe [22] with the factorial Kruskal-Wallis test (*P* < 0.05); the logarithmic LDA threshold score was set at 2.0. A correlation matrix was generated using a pairwise Spearman’s rank correlation coefficient between the top 20 most abundant genera. Only correlations with a statistically significant value (*P* < 0.05) were marked with an asterisk symbol. The black-colored asterisk represents significant correlations with adjusted *P* value (Benjamini-Hochberg false discovery rate (FDR) correction, *P* < 0.05). Finally, the heatmap was visualized with pheatmap package (version 1.10.12). Functional profiling of microbial community was examined by PICRUSt2 (version 2.3.0-b) with picrust2_pipeline.py script based on the KO number and collapsed into pathway-level according to the KEGG database (https://www.kegg.jp/). LEfSe was used to discriminate pathway abundances between groups (LDA score > 2).

### Quantitative PCR of *Akkermansia* and *Turicibacter*

Specific-primer sets targeting the V3-V4 hypervariable region of *Akkermansia* or *Turicibacter* (Additional file 2, Table S1) were designed to quantify the absolute abundance in the samples. To generate standard curves, the type strain for each genus, *A. muciniphila* (KCTC 15667) and *T. sanguinis* (DSM 14220), was prepared as a serially diluted inoculum with various concentrations of cells (10^5^ to 10^8^ cells/ml). The concentration of bacterial cells was measured by QUANTOM Tx™ Microbial Cell Counter (Logos Biosystems, Seoul, Korea). Each inoculum was spiked into 200 mg of sterilized fecal samples and incubated for 2 h at 37 °C. Total DNA was extracted with Fast DNA SPIN kit for fecal samples (MP Biomedicals). Cycle threshold (*C*_T_) values against the logarithm of bacterial cell counts were estimated by the following conditions. Technical triplicated samples were amplified in 10 μl of reaction volume consisting of 5 μl 2 × SYBR Green PCR Master Mix (BioRad Laboratories, Hercules, CA, USA), 1 μl of forward and reverse primers, 4 μl of extracted genomic DNA using QuantStudio 3 Real-Time PCR System (Applied Biosystems, Foster City, CA, USA). The thermal cycling conditions encompassed initial steps (50 °C for 2 min and 95 °C for 10 min) and 40 cycles of extension (95 °C for 15 s and 61 °C for 1 min) using Akk_1-F and Akk_1-R for *A. muciniphila*, Turi_2-F, and Turi_2-R for *T. sanguinis*. Additionally, the final reaction consisting of 95 °C for 15 s, 61 °C for 60 s, and 95 °C for 15 s was performed for melting curve analysis to distinguish the targeted and non-targeted PCR.

### Statistical analysis

The animal studies data were statistically analyzed in Prism (version 8.0.2, Graph Pad Software, Inc.) and results are presented as means with standard deviation (mean ± SD). Data from animal studies were evaluated using a one-way or two-way ANOVA followed by Tukey’s multiple comparison post-test or Bonferroni’s multiple comparison test if significant differences were observed. Unpaired, two-tailed *t*-tests for single comparisons or Wilcoxon rank-sum test were used to assess the significance of the differences in western blot data or bleeding score data. Kaplan-Meier analysis and Log-rank test were used to analyze the survivability. A PERMANOVA test with 999 permutations was used to test group dissimilarity using the Adonis function in the vegan R package.

## Supplementary Information

**Additional file 1: Figure S1.** Construction and validation of the *alr* knockout mutant and *alr* complementation strain. **Figure S2.** Measurement of tumor weights and immunodetection of cell cycle pathway factors in the DLD-1 xenograft mouse model. **Figure S3.** Effects of lyophilized form of PP*-P8 with on polyposis in the AOM/DSS mouse model. **Figure S4.** Principal coordinate analysis based on the Bray-Curtis dissimilarity. **Figure S5.** LEfSe analysis between PP*-P8 and 5-FU.

**Additional file 2: Table S1.** Primer sequences used in this study. **Table S2.** Tumor measurements in the DLD-1 xenograft mouse model. **Table S3.** Statistical analysis of the DLD-1 xenograft mouse results. **Table S4.** Measurements in the AOM/DSS mouse model. **Table S5.** Statistical analysis of the AOM/DSS mouse results. **Table S6.** Metadata of the samples used for 16s rDNA sequencing. **Table S7.** Relative abundances of individual ASVs in each sample.

## Data Availability

The nucleotide sequences used in this study have been deposited in GenBank under the BioProject number PRJNA610226, which comprises 84 Sequence Read Archive files for pyrosequencing reads of the 16S rRNA gene for the fecal microbiotas of AOM/DSS-induced CRC mice (https://www.ncbi.nlm.nih.gov/bioproject/610226).

## Abbreviations

CRC: Colorectal cancer
AOM: Azoxymethane
DSS: Dextran sodium sulfate
LABs: Lactic acid bacteria
DDS: Drug delivery system
d-Ala: d-alanine
PK: Pyruvate kinase
PP-P8: Wild-type *P. pentosaceus* SL4 (-7) with the PK-PK promotor system in pCBT24-2
PP*-P8: Δ*alr* mutant with the PK-PK promotor system in the *alr* vector
PP WT: Wild-type *P. pentosaceus* SL4
ASV: Amplicon sequence variant
PCoA: Principal coordinates analysis
FDR: False discovery rate
LDA: Linear discriminant analysis
LEfSe: Linear discriminant analysis and effect size
MRS: Man, Rogosa, and Sharpe
RPMI: Roswell Park Memorial Institute
TBS: Tris-Buffered-Saline Buffer
TBS-T: Tris-Buffered-Saline Buffer with 0.05% Tween 20
PBS: Phosphate-buffered saline
FBS: 5% fetal bovine serum
GAPDH: Glyceraldehyde 3-phosphate dehydrogenase
PERMANOVA: Permutational multivariate analysis of variance
