# Supplementary material for: A synthetic probiotic engineered for colorectal cancer therapy modulates gut microbiota
Source: Microbiome. 2021 May 26;9:122. doi: 10.1186/s40168-021-01071-4 (PMC8157686; doi:10.1186/s40168-021-01071-4)
Supplement: Supplementary file 2 — Additional file 1: Figure S1. Construction and validation of the alr knockout mutant and alr complementation strain. Figure S2. Measurement of tumor weights and immunodetection of cell cycle pathway factors in the DLD-1 xenograft mouse model. Figure S3. Effects of lyophilized form of PP*-P8 with on polyposis in the AOM/DSS mouse model. Figure S4. Principal coordinate analysis based on the Bray-Curtis dissimilarity. Figure S5. LEfSe analysis between PP*-P8 and 5-FU. [file 40168_2021_1071_MOESM2_ESM.pdf]

A Synthetic Probiotic Engineered for Colorectal Cancer Therapy Modulates Gut Microbiota

Yusook Chung[1,2], Yongku Ryu[1], Byung Chull An[1], Yeo-Sang Yoon[1], Oksik Choi[1], Tai Yeub Kim[1], Jae Kyung Yoon[2], Jun Young Ahn[1], Ho Jin Park[1], Soon-Kyeong Kwon[2,3], Jihyun F. Kim[2,†], and Myung Jun Chung[1,†]

[1]R&D Center, Cell Biotech, Co., Ltd., 50 Aegibong-ro 409beon-gil, Gaegok-ri, Wolgot-myeon, Gimpo-si, Gyeonggi-do 10003, Republic of Korea; [2]Department of Systems Biology, Division of Life Sciences, and Institute for Life Science and Biotechnology, Yonsei University, 50 Yonsei-ro, Seodaemun-gu, Seoul 03722, Republic of Korea; [3]Division of Life Science, Gyeongsang National University, 501 Jinju-daero, Jinju-si, Gyeongsangnam-do 52828, Republic of Korea

[*]Corresponding authors:

Myung Jun Chung; phone: +82-31-987-6205, fax: +82-31-987-6216, e-mail: ceo@cellbiotech.com

Jihyun F. Kim; phone: +82-2-2123-5561, fax: +82-2-312-5657, e-mail: jfk1@yonsei.ac.kr

SUPPLEMENTARY FIGURE LEGENDS

Supplementary Figure 1 Construction and validation of the *alr* knockout mutant and *alr* complementation strain.

(A) General scheme of the *alr* complementation system construction. (B) Confirmation of the *alr* knockout mutant and *alr* complemented strains cultured in MRS media with or without D-Ala. (C) Genotyping of the *alr* gene in the wild-type and *alr* knockout mutant strains. (D) Comparison of the secreted form of P8 between wild-type and *alr* knockout mutant strains. Recombinant form of P8.

Supplementary Figure 2 Measurement of tumor weights and immunodetection of cell cycle pathway factors in the DLD-1 xenograft mouse model

(A) Tumor weights measured at week 6. Mice (n = 10 in each group) were subcutaneously inoculated with 2×10^6 DLD-1 cells in the rear right flank and then received 0.9% saline (control), 60 mg/kg body weight gemcitabine (dFdC; intraperitoneal injection, twice a week), 1×10^{10} CFU/head *P. pentosaceus alr* (pCBT24-2-alr) (PP*; oral administration, five times a week), or 1×10^{10} CFU/head *P. pentosaceus alr* (pCBT24-2-PK-p8-PK-p8-alr) (PP*-P8; oral administration, five times a week). ***P < 0.001 (B) Immunodetection of cell cycle regulatory factors in untreated control, PP*, and PP*-P8.

Supplementary Figure 3 Effects of lyophilized form of PP*-P8 with on polyposis in the AOM/DSS mouse model

Number of polyps were measured after 68 days. Treatment groups: untreated control (0.9% saline), *P. pentosaceus alr* (pCBT24-2-PK-p8-PK-p8-alr) (PP*-P8; 1×10^{10} CFU/head) and *P. pentosaceus alr* (pCBT24-2-PK-p8-PK-p8-alr) (PP*-P8-L; 10 x lyophilized of PP*-P8). ***P* < 0.01

Supplementary Figure 4 Principal coordinate analysis based on the Bray-Curtis dissimilarity. Each dot indicates a single sample and each group are shown in a different color. *P*-values correspond to the results of permutational multivariate analysis of variance. Day 0 and Day 5 samples were included in the all stages to observe longitudinal sample dissimilarity.

Supplementary Figure 5 LEfSe analysis between PP*-P8 and 5-FU.

(A) Linear discriminant analysis effect size of samples. Treatments: *P. pentosaceus alr* (pCBT24-2-alr)(PP*-P8; 1×10^{10} CFU/head), 5-FU, fluorouracil. (B) Taxonomic cladogram results from linear discriminant analysis (LDA) effect size. Red, taxa enriched in 5-FU; green, taxa enriched in PP*-P8.

SUPPLEMENTARY FIGURES

Figure S1

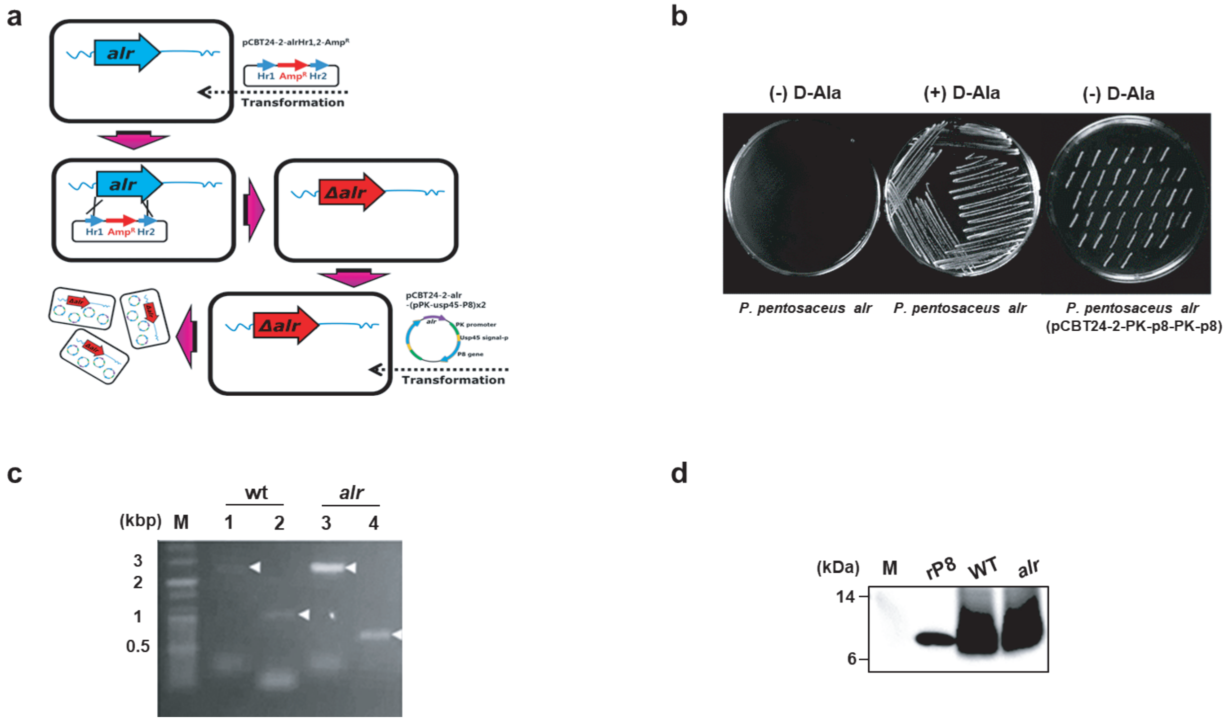

Figure S2

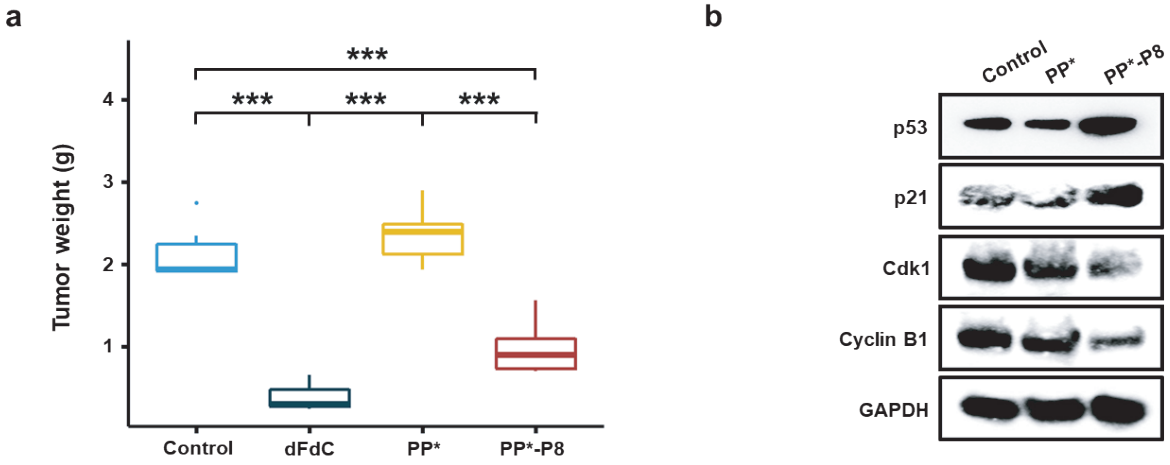
a
***
*** *** ***
4
3
2
1
Tumor weight (g)
Control
dFdC
PP*
PP*-P8
b
Control
PP*
PP*-P8
p53
p21
Cdk1
Cyclin B1
GAPDH

Figure S3

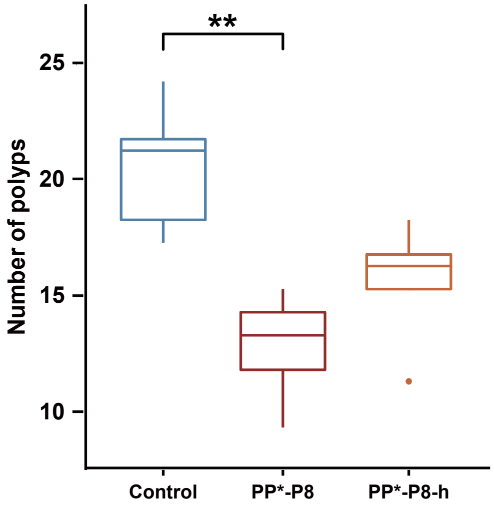

Figure S4

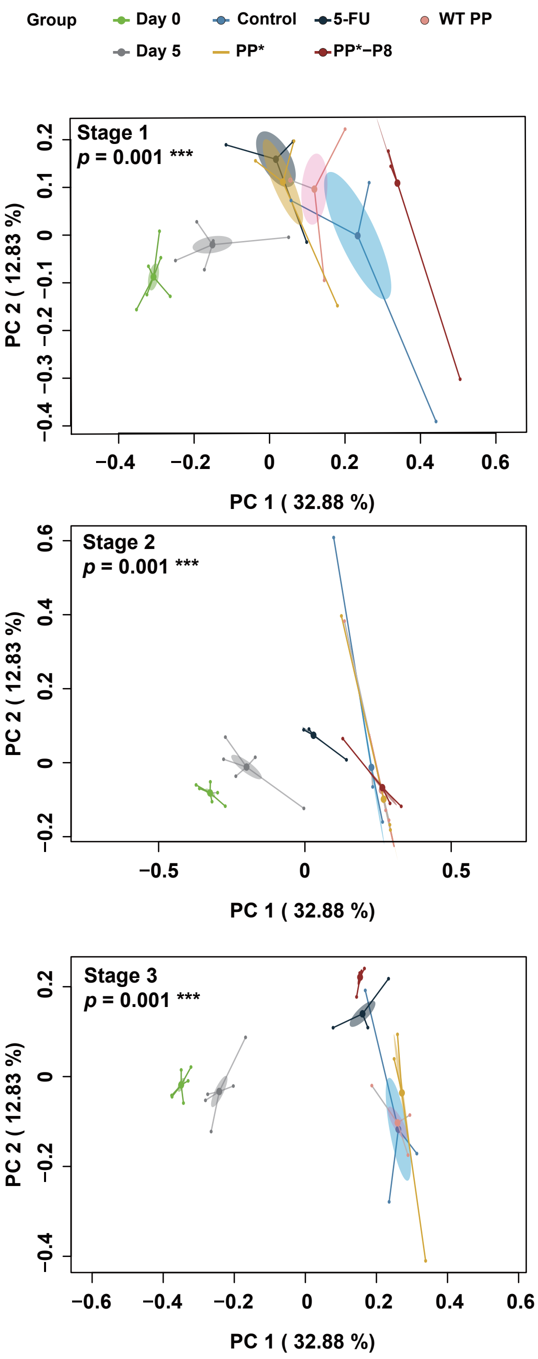
Group
Day 0
Control
5-FU
WT PP
Day 5
PP*
PP*−P8
Stage 1
p = 0.001 ***
PC 2 (12.83 %)
PC 1 (32.88 %)
Stage 2
p = 0.001 ***
Stage 3
p = 0.001 ***

Figure S5

a

b

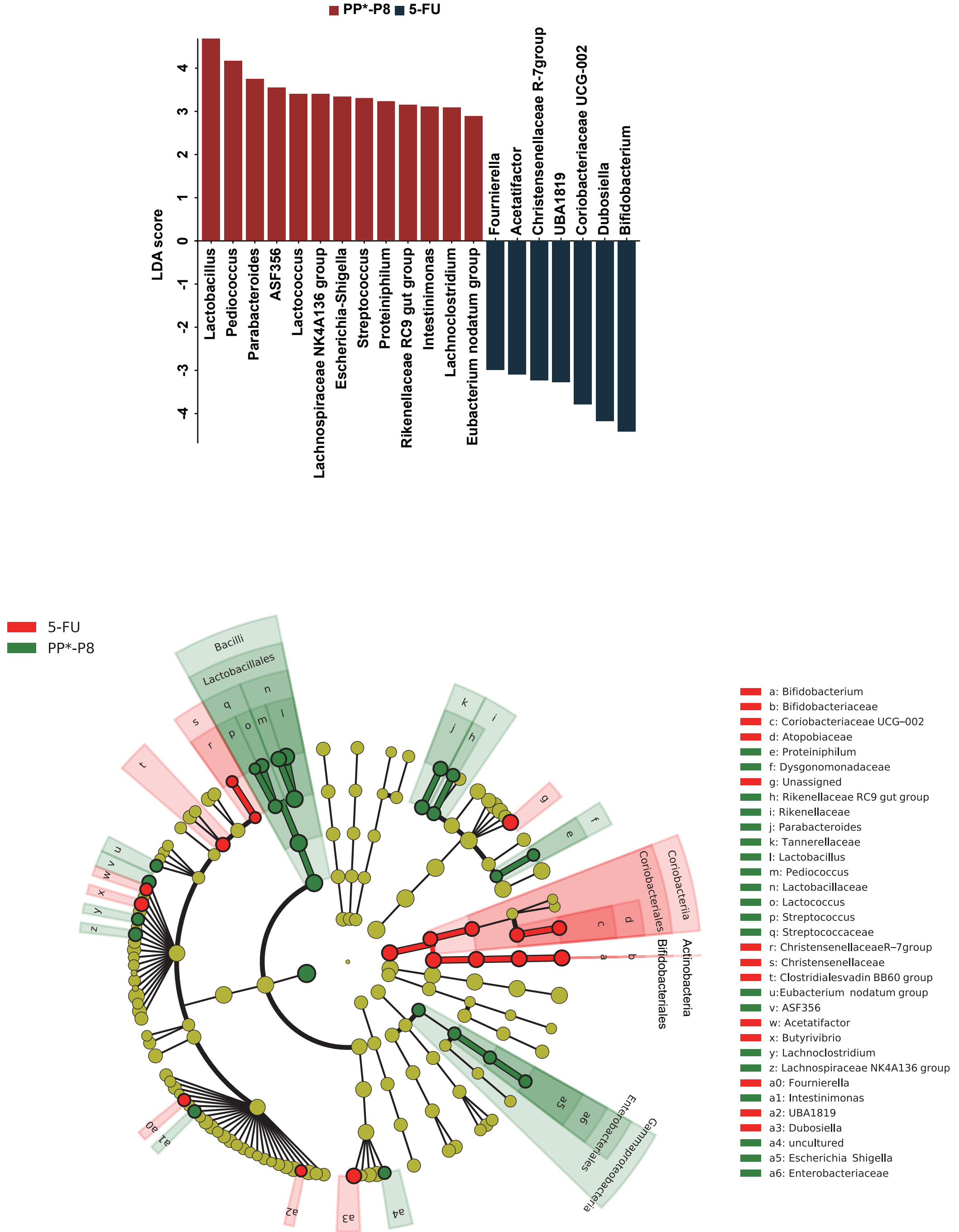

PP*-P8
5-FU
LDA score
4
3
2
1
0
-1
-2
-3
-4
Lactobacillus
Pediococcus
Parabacteroides
ASF356
Lactococcus
Lachnospiraceae NK4A136 group
Escherichia-Shigella
Streptococcus
Proteiniphilum
Rikenellaceae RC9 gut group
Intestinimonas
Lachnoclostridium
Eubacterium nodatum group
Fournierella
Acetatifactor
Christensenellaceae R-7group
UBA1819
Coriobacteriaceae UCG-002
Dubosiella
Bifidobacterium
5-FU
PP*-P8
Bacilli
Lactobacillales
Actinobacteria
Coriobacteriia
Coriobacteriales
Bifidobacteriales
Enterobacteriales
Gammaproteobacteria
a: Bifidobacterium
b: Bifidobacteriaceae
c: Coriobacteriaceae UCG–002
d: Atopobiaceae
e: Proteiniphilum
f: Dysgonomonadaceae
g: Unassigned
h: Rikenellaceae RC9 gut group
i: Rikenellaceae
j: Parabacteroides
k: Tannerellaceae
l: Lactobacillus
m: Pediococcus
n: Lactobacillaceae
o: Lactococcus
p: Streptococcus
q: Streptococcaceae
r: ChristensenellaceaeR–7group
s: Christensenellaceae
t: Clostridialesvadin BB60 group
u:Eubacterium nodatum group
v: ASF356
w: Acetatifactor
x: Butyrivibrio
y: Lachnoclostridium
z: Lachnospiraceae NK4A136 group
a0: Fournierella
a1: Intestinimonas
a2: UBA1819
a3: Dubosiella
a4: uncultured
a5: Escherichia Shigella
a6: Enterobacteriaceae
